# Agarwood wound locations provide insight into the association between fungal diversity and volatile compounds in *Aquilaria sinensis*

**DOI:** 10.1098/rsos.190211

**Published:** 2019-07-03

**Authors:** Juan Liu, Xiang Zhang, Jian Yang, Junhui Zhou, Yuan Yuan, Chao Jiang, Xiulian Chi, Luqi Huang

**Affiliations:** 1 National Resource Center for Chinese Materia Medica, China Academy of Chinese Medical Sciences, Beijing 100700, People's Republic of China; 2 Department of Traditional Chinese Medicine, Guangdong Pharmaceutical University, Guangzhou 510006, People's Republic of China

**Keywords:** agarwood, *Aquilaria sinensis*, fungal community, fungal diversity, two-dimensional gas chromatography with high-resolution time-of-flight mass spectrometry, volatile compound

## Abstract

The aim of the present study was to investigate the effect of wound location on the fungal communities and volatile distribution of agarwood in *Aquilaria sinensis*. Two-dimensional gas chromatography with high-resolution time-of-flight mass spectrometry revealed 60 compounds from the NIST library, including 25 sesquiterpenes, seven monoterpenes, two diterpenes, nine aromatics, nine alkanes and eight others. Of five agarwood types, Types IV and II contained the greatest number and concentration of sesquiterpenes, respectively. The fungal communities of the agarwood were dominated by the phylum Ascomycota and were significantly affected by the type of wound tissue. Community richness indices (observed species, Chao1, PD whole tree, ACE indices) indicated that Types I and IV harboured the most and least species-rich fungal communities, and the fungal communities of Types V, I, III and IV/II were dominated by *Lasiodiplodia*, *Hydnellum*, *Phaeoisaria* and *Ophiocordyceps* species, respectively. Correlations between fungal species and agarwood components revealed that the chemical properties of *A. sinensis* were associated with fungal diversity. More specifically, the dominant fungal genera of Types V, I and III (*Lasiodiplodia*, *Hydnellum* and *Phaeoisaria*, respectively) were strongly correlated with specific terpenoid compounds. The finding that wound location affects the fungal communities and volatile distribution of agarwood provides insight into the formation of distinct agarwood types.

## 1. Introduction

Agarwood, also known as ‘wood of the Gods’, is of huge cultural significance, due to its peculiar perfume and use in incense ceremonies. Derived from the resinous portions of trunks and branches from *Aquilaria* and *Gyrinops* species, it is the basis of some of the most world's most exclusive perfumes and is used extensively for medicine and incense across Asia, the Middle East and Europe [1,2]. This widespread use can likely be attributed to the sesquiterpene and phenylethyl chromone derivatives of agarwood [3–5], which have biological and pharmacological properties, including antimicrobial, anti-oxidant and anti-proliferative activities [6–8]. Moreover, agarwood has the potential to prevent cancer and to treat both gastric ulcers and cough in asthma patients [8–10]. Indeed, in China, agarwood, which is mainly derived from *Aquilaria sinensis* (Lour.) Spreng, is valued as a traditional Chinese medicine for the treatment of emesia, asthma and insomnia [11,12]. However, wild sources of agarwood are at serious risk of depletion, owing to the slow and infrequent formation of agarwood and to the resource's uncontrolled collection in forests. As a result, all *Aquilaria* and *Gyrinops* species are endangered and are listed in Appendix II of the Convention on International Trade in Endangered Species of Wild Fauna and Flora (CITES, http://www.cites.org). To protect both wild agarwood resources and sustainable agarwood production, *A. sinensis* has been widely cultivated in Guangdong and Hainan provinces in China, and due to its rarity and value, *A. sinensis* has also been used to investigate processes for improving the oil yield of agarwood in China.

Under natural conditions, agarwood is produced when attacked by microbes, insects or other damaging organisms and promotes the accumulation of agarwood resin [13]. Fungal infection has long been recognized as the cause of agarwood formation in *Aquilaria* host trees [14–16]; a variety of fungi (e.g. *Fusarium* sp., *Chaetomium globosum*, *Menanotus flavolives*, *Lasiodiplodia theobromae* and *Rigidoporus vinctus*) isolated from infectious *Aquilaria* trees have been reported to accelerate agarwood formation and to promote the accumulation of volatile compounds [2,17–21]. Agarwood is categorized, in China, into different types on the basis of the location at which it forms [22,23]. However, even though the various agarwood types are widely traded on the Chinese market, the relationship between the volatile compounds and fungal diversity of agarwood remains relatively unclear. Elucidating such relationships would help to delineate the numerous potential fungal niches and chemical characteristics of agarwood formed in different regions of the trees.

Using *A. sinensis* as a model system for agarwood formation, the aim of the present study was to investigate the variation of fungal communities across habitats within a tree host from different agarwood-formed tissues. Comprehensive two-dimensional gas chromatography with high-resolution time-of-flight mass spectrometry (GC × GC-HR-TOF-MS), which is a versatile analytical tool combining two powerful analytical technologies with complementary attributes [24,25], was also used to elucidate the volatile constituents of different types of agarwood. In addition, the regular patterns of fungal communities and volatile constituents observed in different types of agarwood facilitated the characterization of fungal community structures within different parts of agarwood formation, as well as the investigation of the relationship between fungal diversity and volatile compound production, which could help elucidate the variation of agarwood fragrance.

## 2. Material and Methods

### 2.1. Study location and sampling methods

**Figure 1.**
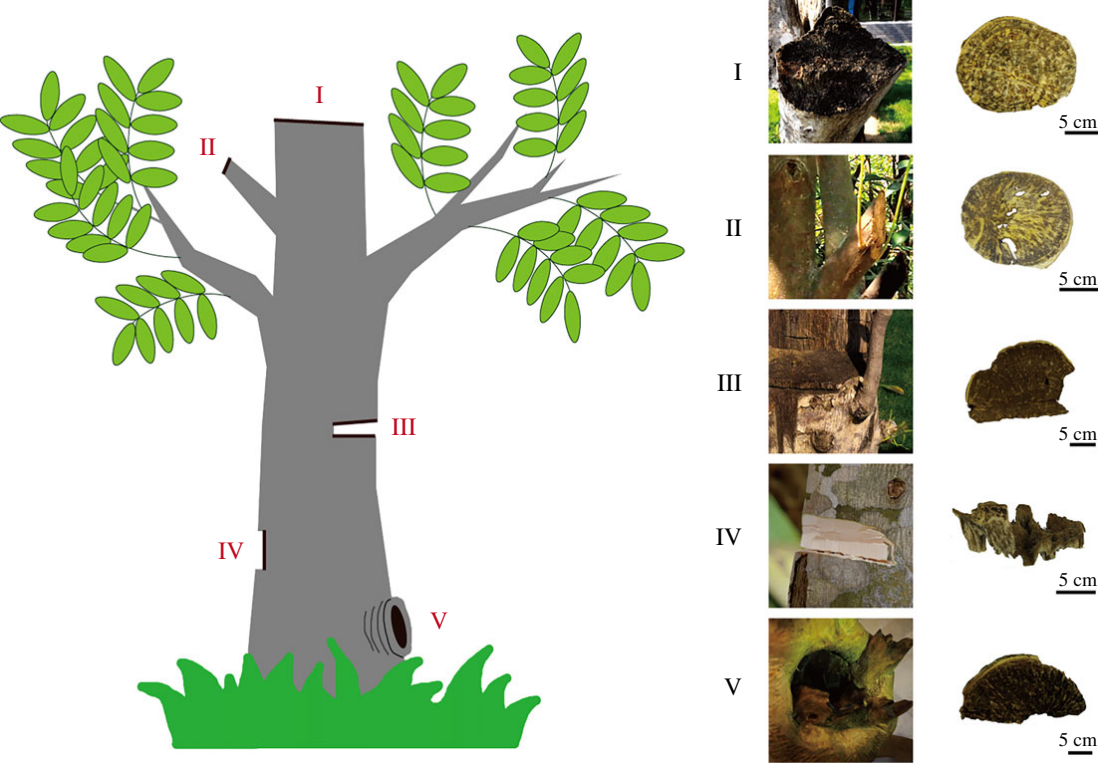
Wound tissues and agarwood sample collection from *Aquilaria sinensis.*

Thirty-year-old *A. sinensis* trees in Dalingshan town, Guangdong province, China (latitude 22°45′43″ N, longitude 113°48′45″ E), were physically wounded using a machete according to the previous treated method [26,27], and after five years, 0.5- to 1-cm thick agarwood sections had formed beneath the wounded surface. In November 2017, the agarwood was collected by cutting at about 5 cm below the wound area, and the non-agarwood parts were removed from the samples. These trees were similar in diameter, about 20–30 cm and spaced at intervals of about 5–10 m. Fifteen trees were wounded at different locations as following, with each treatment group including three individual trees. The five types of agarwood that formed at the different wound points were considered Type I, II, III, IV and V, respectively (figure 1). Type I agarwood formed in the transverse section of wounded main trunks at about 2.0–2.5 m above the ground. Type II agarwood formed in the transverse section of wounded lateral branches at about 1.5–1.8 m above the ground. Type III agarwood formed in the transverse section of wounded main trunks at about 1.0–1.2 m above the ground. Type IV formed in the longitudinal section of main trunks at about 0.8–1.2 m above the ground, and Type V usually formed in the main trunks near the root, which was encapsulated by surrounding tissue (at about 0.2–0.4 m above the ground).

### 2.2. DNA extraction, PCR amplification and DNA sequencing

All agarwood samples were washed with distilled sterile water and were surface sterilized by soaking in 75% ethanol for 2 min, after which they were rinsed with sterile water and dried on sterile filter paper. Total DNA was extracted from each agarwood sample (100 mg) using the MoBio PowerPlant Pro DNA Isolation Kit (MoBio Laboratories, Inc., Carlsbad, CA, USA). Two replicate extractions were performed for each agarwood sample, in order to achieve sufficient DNA yields, and DNA quantity was determined using electrophoresis on 1% agarose gels. The extracted DNA was quantified using a NanoDrop 1000 spectrophotometer (NanoDrop Products, Wilmington, DE, USA). According to the concentration, each DNA sample was diluted to 1 ng µl^−1^ using sterile water. Internal transcribed spacer 1 (ITS1) sequences were then amplified from all the samples using the universal primers ITS5-1737F (5′-GGAAGTAAAAGTCGTAACAAGG-3′) and ITS2-2043R (5′-GCTGCGTTCTTCATCGATGC-3′) and barcodes to distinguish between the samples. The PCR was performed using a Phusion High-Fidelity PCR Master Mix (New England Biolabs, Ipswich, MA, USA), according to Cregger *et al.* [28]. The resulting PCR products were mixed in equal ratios and purified using a Qiagen Gel Extraction Kit (Qiagen, Duesseldorf, Germany). Sequencing libraries were then generated using the Ion Plus Fragment Library Kit (Thermo Fisher Scientific, Waltham, MA, USA), following the manufacturer's recommendations, and index codes were added. Library quality was assessed using a Qubit v. 2.0 Fluorometer (Thermo Fisher Scientific) and an Agilent Bioanalyzer 2100 system (Agilent Technologies, Inc., Palo Alto, CA, USA), and the library was sequenced using a Thermofisher Life Ion S5 platform (Thermo Fisher Scientific). The high-throughput sequencing data are available from the National Center for Biotechnology Information (NCBI) database in the Sequence Read Archive (SRA) database, under BioProject number PRJNA509099.

### 2.3. Bioinformatics processing

Raw sequences (2 × 300-bp reads) were generated using an Illumina MiSeq system (Illumina, San Diego, CA, USA) and MiSeq reagent kit v. 3 (Illumina) by the University of Wisconsin Biotechnology Center (Madison, WI, USA) [29]. Low-quality reads were first removed using Cutadapt v. 1.9.1 (http://cutadapt.readthedocs.io/en/stable/), and paired-end reads were categorized according to the unique barcodes that were then removed along with the primer sequences. Overlapping reads were merged using FLASH [30], and high-quality clean tags were filtered using the QIIME quality-control process [31]. The tags were compared with the Unite database [32], using the UCHIME algorithm to remove chimaeric sequences and to obtain clean reads [33]. High-quality sequences were clustered into operational taxonomic units (OTUs), which were defined as 97% similar, using the UPARSE software [34]. These OTUs were applied to analyse diversity, richness and rarefaction curves using MOTHUR [35]. Taxonomic assignments of OTUs that achieved 97% similarity were obtained using the QIIME v. 1.9.1 [31] software package through comparison with the SILVA [36], Greengene [37] and RDP [38] databases. Subsequent analyses on alpha and beta diversity were performed based on the normalized OTU abundance. Venn diagrams, alpha diversity (including Chao1 and ACE richness estimators, Shannon and Simpson diversity indices, and phylogenetic diversity whole tree), beta diversity (including principal co-ordinates analysis, principal component analysis and non-metric multidimensional scaling) and heat map analysis were performed to identify mutual and unique taxa between groups, and the above analysis of fungal species diversity in samples using R software (http://www.r-project.org/).

### 2.4. Extraction of volatile oil

Dried agarwood powder (0.1 g) from each sample was separately weighed and placed in a 2-ml centrifuge tube, and 1.5 ml ethyl acetate was added to the tube. The powder was soaked overnight at room temperature and extracted for 45 min using the 40-kHz ultrasonic cold extraction method, according to Liao *et al.* [39] with slight modification. The solvent phase (upper layer) was separated by centrifugation at 12 000 r.p.m. and 4°C for 10 min. After adding ethyl acetate to supplement the reduced weight, the volatile oil was filtered through a 0.22-µm PTFE filter membrane and then stored in a non-transparent glass bottle at 4°C prior to GC × GC-HR-TOF-MS analysis.

### 2.5. GC × GC-HR-TOF-MS analysis

The GC × GC-HR-TOF-MS system consisted of an East & West 3300 GC × GC equipped with a TOF-MS (East & West Analytical Instrument Co., Beijing, China), which is used to acquire mass spectral data from the GC × GC. The first separation was performed in a conventional non-polar GC column Agilent DB-5MS (30 m × 0.25 mm inner diameter × 0.25 µm film thickness), and the second was performed in a medium GC column Agilent DB-17HT (2.5 m × 0.25 mm inner diameter × 0.15 µm film thickness). An aliquot (1 µl) of each sample was separately injected into the GC injector, using a split ratio of 30 : 1 at 300°C. The separation was performed under the following conditions: initial temperature of 70°C for 1 min, ramped at 6°C min^−1^ to 300°C, and held at 300°C for 1 min. The transfer line into the TOF-MS source was heated to 270°C, and the electron impact ionization source was operated at 240°C with a collision energy of 70 eV and an acquisition voltage of 1800 V. The mass spectrometer was operated at an acquisition rate of 100 spectra s^−1^, ranging from 50 to 550 u. The relative contents of the individual components of each sample were expressed in percentage of peak area relative to the total peak area. The identification of volatiles of the agarwood samples was based on a National Institute of Standards and Technology 11 (NIST11) library search combined with the Kovats retention index (RI) [40]. Compounds with lower search probabilities (less than 60) were regarded as unknowns. The mass spectral match factor (probability > 60) was used to judge whether a peak was correctly identified or not. For the determination of RI, calculated on the first dimension, a series of n-alkanes (C9–C23) was used under the same experimental conditions. The RI for each compound was calculated as follows:

$$\mathrm{RI}=100\times \left\{n+\frac{\log t^{\prime }(i)-\log t^{\prime }(n)}{\log t^{\prime }(n+1)-\log t^{\prime }(n)}\right\},$$

where *n* and *n +* 1 are the number of carbon atoms in alkanes eluting before and after the compound, respectively; *t′*(*n*) and *t′*(*n + 1*) were the corresponding retention time values and *t′*(*i*) was the retention time of the identified compound.

### 2.6. Statistical analysis

The mean and standard error (s.e.) of all data were calculated, and all values were reported as the mean of three replicates. The community richness and diversity indices of the fungi and volatile contents of the five types of agarwood were analysed using one-way analysis of variance (ANOVA), with a significance level of *p* < 0.05. Heat map analysis was used to analyse the abundance of fungal distribution and the concentration of volatile compounds. Principal component analysis (PCA) was also performed to investigate the differences in volatile contents among the above agarwood samples. In addition, Pearson correlation analysis was used to investigate the relationship between fungal communities and volatile components. All statistical analyses were performed using SPSS v. 16.0 (SPSS, Inc., Armonk, NY, USA) [41].

## 3. Results

### 3.1. Identification of characterized agarwood components

**Figure 2.**
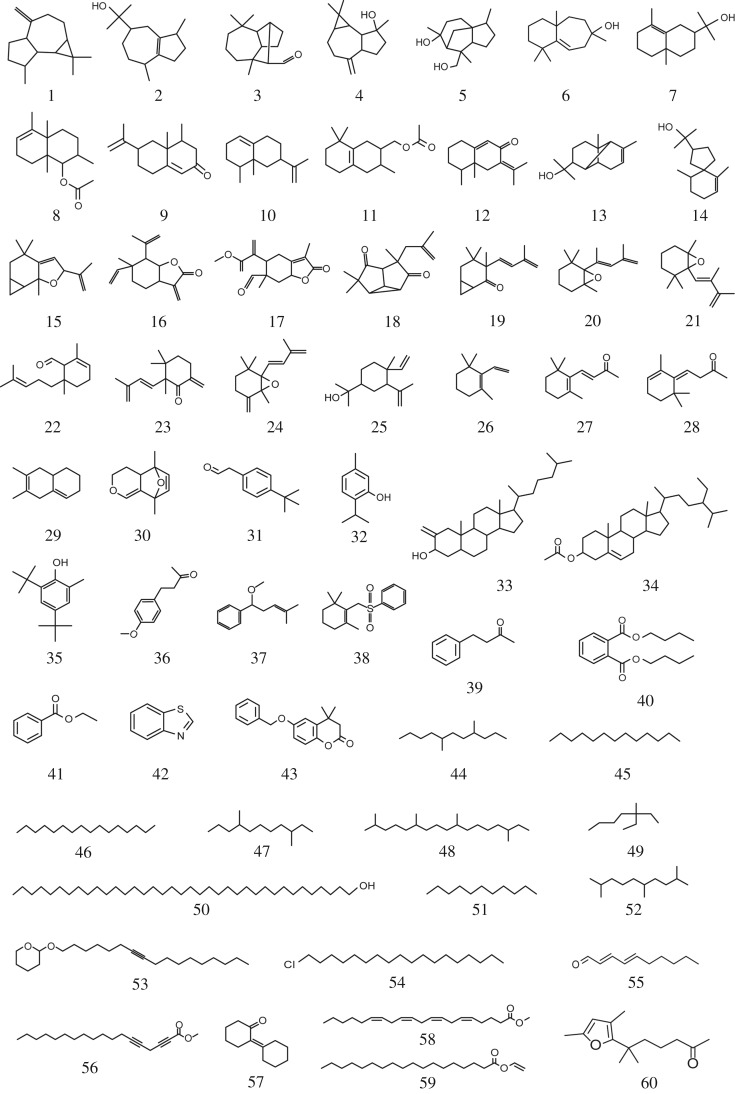
Structures of volatile compounds identified in the agarwood samples using GC × GC-HR-TOF-MS. Compound numbers follow the nomenclature used in table 1.

To investigate the relationship between agarwood characteristics and the presence of specific fungal species, the volatile compounds of different agarwood types were first identified using GC × GC-HR-TOF-MS. More specifically, the volatile compounds of five different agarwood samples were separated and identified using a DB-5MS column on the first dimension and a DB-17HT column on the second dimension via GC × GC-HR-TOF-MS analysis. This analysis identified 60 strong matches to the NIST library data, including 25 sesquiterpenes, seven monoterpenes, two diterpenes, nine aromatics, nine alkanes and eight other components (table 1 and figure 2). The agarwood volatile fraction was characterized by a high percentage of sesquiterpenes, including guaianes (compounds 1–5; figure 2), eudesmanes (compounds 7–13; figure 2), agarofurans (compound 14; figure 2) and several others (compounds 6, 15–25; figure 2). Of these sesquiterpenes, two compounds, namely α-eudesmol and α-copaen-11-ol, were found in all the agarwood samples (electronic supplementary material, figure S1, and table 1).

**Table 1.** Volatile compounds identified in the agarwood samples using GC×GC-HR-TOF-MS. Library probability of each compound was above 60 Mol. Wt., molecular weight. CAS No., library CAS number.

no.	compound name	RI	peak I/min	peak II/s	library formula	library probability	CAS No.	Mol. Wt.	I (%)	II (%)	III (%)	IV (%)	V (%)
	*sesquiterpenes*												
1	aromadendrene	1533	12.72	3.27	C_15_H_24_	82	489394	204	0.0015 ± 0.0010	not detected	not detected	0.6923 ± 0.5293	not detected
2	guaiol	1663	15.28	5.16	C_15_H_26_O	79	489861	222	5.3112 ± 3.7519	not detected	not detected	2.3544 ± 0.1645	4.2907 ± 3.8226
3	longifolenaldehyde	1497	12.48	3.55	C_15_H_24_O	73	19890847	220	not detected	not detected	not detected	1.3292 ± 0.9446	not detected
4	espatulenol	1497	12.48	2.52	C_15_H_24_O	76	6750603	220	1.7892 ± 0.1246	2.3159 ± 1.7728	not detected	1.1377 ± 0.9486	1.1186 ± 0.6457
5	8-(hydroxymethyl)-3,6,8-trimethyloctahydro-1H-3a,7-methanoazulen-6-ol	1975	20.77	0.37	C_15_H_26_O_2_	73	62600059	238	not detected	not detected	0.7556 ± 0.6302	0.0354 ± 0.0190	0.0409 ± 0.0230
6	1,1,4a,7-tetramethyl-2,3,4,4a,5,6,7,8-octahydro-1H-benzo[7]annulen-7-ol	2140	26.13	2.57	C_15_H_26_O	68	6892804	222	not detected	0.0759 ± 0.0203	not detected	not detected	not detected
7	α-eudesmol	1634	14.82	4.24	C_15_H_26_O	83	1209718	222	0.7001 ± 0.2812	0.1698 ± 0.0071	0.9775 ± 0.1515	1.0196 ± 0.6624	0.7444 ± 0.6523
8	2,4a,5,8a-tetramethyl-1,2,3,4,4a,7,8,8a-octahydronaphthalen-1-yl acetate	1712	16.1	5.27	C_16_H_26_O_2_	69	20558218	250	5.5941 ± 1.6811	not detected	not detected	6.8546 ± 3.4922	11.0327 ± 9.7659
9	5,6-dimethyl-8-isopropenylbicyclo(4.4.0)dec-1-en-3-one	1744	16.68	3.77	C_15_H_22_O	69	4674504	218	not detected	3.2207 ± 2.8745	not detected	not detected	not detected
10	(+)-valencene	1511	12.72	2.45	C_15_H_24_	81	4630073	204	not detected	0.1616 ± 0.0175	not detected	not detected	not detected
11	(3,8,8-trimethyl-1,2,3,4,5,6,7,8-octahydro-2-naphthalenyl)methyl acetate	1719	16.22	0.61	C_16_H_26_O_2_	68	314773278	250	not detected	not detected	3.6388 ± 0.0790	not detected	7.3226 ± 0.0343
12	dehydrofukinone	1820	18.08	2.38	C_15_H_22_O	78	19598459	218	not detected	3.7005 ± 2.7568	not detected	0.6524 ± 0.1635	not detected
13	α-copaen-11-ol	1685	15.63	5.89	C_15_H_24_O	73	41370563	220	5.2734 ± 1.7874	7.1112 ± 5.6153	4.0888 ± 1.5279	1.9115 ± 1.4793	0.0369 ± 0.0147
14	agaruspirol	1663	15.28	4.49	C_15_H_26_O	83	1460737	222	not detected	4.1321 ± 1.6386	not detected	6.2798 ± 3.1912	not detected
15	2-isopropenyl-4,4,6b-trimethyl-4,5,5a,6,6a,6b-hexahydro-2H-cyclopropa[g][1]benzofuran	1646	15.87	4.84	C_15_H_22_O	76	102681492	218	not detected	28.1937 ± 4.0029	not detected	3.6005 ± 0.2307	1.3055 ± 1.0888
16	dehydrosaussurea lactone	2102	24.73	4.71	C_15_H_20_O_2_	74	28290359	232	not detected	1.2429 ± 0.0490	not detected	0.0406 ± 0.0223	0.1199 ± 0.0372
17	methyl-2-(3,6-dimethyl-2-oxo-6-vinyl-2,4,5,6,7,7a-hexahydrobenzofuran-5-yl)acrylate	1867	18.9	3.02	C_16_H_20_O_4_	61	19892194	276	not detected	not detected	not detected	0.2207 ± 0.1593	not detected
18	2,2,4-trimethyl-4-(2-methylallyl)hexahydrocyclopropa[cd]pentalene-1,3-dione	1854	18.67	2.21	C_15_H_20_O_2_	74	94609184	232	0.1986 ± 0.1178	not detected	0.7312 ± 0.1133	0.0796 ± 0.0548	0.1786 ± 0.1174
19	3,4,4-trimethyl-3-[(1E)-3-methyl-1,3-butadienyl]bicyclo[4.1.0]heptan-2-one	1867	18.9	2.72	C_15_H_22_O	69	102146816	218	not detected	not detected	not detected	0.1445 ± 0.0096	not detected
20	(E)-2,2,6-trimethyl-1-(4-methylpenta-2,4-dien-2-yl)-7-oxabicyclo[4.1.0]heptane	2028	22.17	4.51	C_15_H_24_O	68	89128143	220	not detected	not detected	not detected	0.1581 ± 0.0127	0.5011 ± 0.1012
21	1-[(1E)-2,3-Dimethyl-1,3-butadienyl]-2,2,6-trimethyl-7-oxabicyclo[4.1.0]heptane	2028	22.17	5.76	C_15_H_24_O	66	59744126	220	not detected	not detected	not detected	1.5545 ± 0.8055	not detected
22	2,6-dimethyl-6-(4-methyl-3-pentenyl)-2-cyclohexene-1-carbaldehyde	1887	19.25	1.13	C_15_H_24_O	72	56772077	220	not detected	0.9327 ± 0.7353	not detected	not detected	not detected
23	(E)-2,3,3-trimethyl-2-(3-methylbuta-1,3-dien-1-yl)-6-methylenecyclohexan-1-one	2208	28.58	2.65	C_15_H_22_O	71	77822572	218	not detected	not detected	not detected	not detected	1.2203 ± 0.7010
24	(E)-2,2,6-trimethyl-1-(3-methylbuta-1,3-dien-1-yl)-5-methylene-7-oxabicyclo[4.1.0]heptane	2297	31.62	2.06	C_15_H_22_O	70	70038209	218	not detected	not detected	not detected	not detected	1.2714 ± 0.9411
25	2-(3-isopropenyl-4-methyl-4-vinylcyclohexyl)-2-propanol	1678	15.52	1.61	C_15_H_26_O	68	639996	222	not detected	not detected	not detected	not detected	0.4491 ± 0.3673
	total sesquiterpenes								18.8681	51.2570	10.1919	28.0654	29.6327
	*monoterpenes*												
26	1,3,3-trimethyl-2-vinylcyclohexene	1706	15.98	5.27	C_11_H_18_	73	5293903	150	6.7032 ± 4.6849	not detected	0.7544 ± 0.4255	1.8548 ± 0.0363	not detected
27	(E)-b-ionone	1840	18.43	6.25	C_13_H_20_O	66	79776	192	4.8090 ± 4.0616	not detected	not detected	not detected	0.2716 ± 0.2292
28	(4E)-4-(2,6,6-trimethyl-2-cyclohexen-1-ylidene)-2-butanone	2147	26.37	2.38	C_13_H_20_O	66	56052610	192	not detected	not detected	not detected	not detected	1.5186 ± 0.8964
29	6,7-dimethyl-1,2,3,5,8,8a-hexahydronaphthalene	1275	9.1	2.4	C_12_H_18_	74	107914921	162	not detected	0.8786 ± 0.2955	2.0628 ± 0.8246	not detected	not detected
30	1,8-dimethyl-4,11-dioxatricyclo[6.2.1.02,7]undeca-2,9-diene	1591	14.12	5.65	C_11_H_14_O_2_	70	121029638	178	not detected	not detected	1.6647 ± 0.2766	not detected	not detected
31	(4-tert-butylphenyl)acetaldehyde	1424	11.32	4.13	C_12_H_16_O	73	109347457	176	not detected	not detected	not detected	not detected	0.0275 ± 0.0068
32	thymol	1762	17.03	4.09	C_10_H_14_O	70	89838	150	not detected	8.3843 ± 7.7453	not detected	not detected	not detected
	total monoterpenes								11.5122	9.2629	4.4819	1.8548	1.8177
	*diterpenes*												
33	2-methylenecholestan-3-ol	1854	18.67	3.33	C_28_H_48_O	70	22599968	400	0.0810 ± 0.0347	0.0094 ± 0.0030	not detected	not detected	0.0674 ± 0.0061
34	stigmast-5-en-3-yl acetate	1752	17.73	1.66	C_31_H_52_O_2_	71	915059	456	not detected	not detected	not detected	not detected	1.1457 ± 0.4734
	total diterpenes								0.0810	0.0094	0.0000	0.0000	1.2131
	*aromatics*												
35	4,6-di-tert-butyl-o-cresol	1490	12.37	1.67	C_15_H_24_O	68	616557	220	0.1980 ± 0.0642	not detected	0.6796 ± 0.1222	0.5806 ± 0.0178	0.7009 ± 0.1337
36	p-methoxybenzylacetone	1524	12.95	3.5	C_11_H_14_O_2_	80	104201	178	not detected	0.4269 ± 0.0940	not detected	0.1421 ± 0.0612	0.4741 ± 0.2739
37	(1-methoxy-4-methyl-3-pentenyl)benzene	1921	19.83	1.91	C_13_H_18_O	72	68705862	190	not detected	0.2839 ± 0.2257	2.4052 ± 1.6184	3.3340 ± 0.4246	1.4515 ± 0.3223
38	(2,6,6-trimethylcyclohex-1-enylmethanesulfonyl) benzene	2048	22.87	2.51	C_16_H_22_O_2_S	68	56691748	278	not detected	not detected	not detected	0.2523 ± 0.0559	0.7627 ± 0.2472
39	4-phenylbutan-2-one	1267	8.98	2.3	C_10_H_12_O	86	2550267	148	not detected	0.4726 ± 0.1153	not detected	3.5987 ± 2.6857	not detected
40	dibutyl phthalate	1961	20.53	5.83	C_16_H_22_O_4_	74	84742	278	7.3164 ± 4.8192	not detected	not detected	not detected	not detected
41	ethyl benzoate	1195	7.93	2.01	C_9_H_10_O_2_	85	93890	150	not detected	0.5335 ± 0.1159	not detected	not detected	not detected
42	benzothiazole	1259	8.87	2.79	C_7_H_5_NS	83	95169	135	not detected	0.2800 ± 0.0481	not detected	not detected	not detected
43	6-(benzyloxy)-4,4-dimethylchroman-2-one	1627	14.7	2.28	C_18_H_18_O_3_	72	84945108	282	not detected	0.2019 ± 0.0437	not detected	not detected	not detected
	total aromatics								7.5144	2.1988	3.0848	7.9077	3.3892
	*alkanes*												
44	4,7-dimethylundecane	1065	6.07	1.2	C_13_H_28_	85	17301325	184	not detected	0.7911 ± 0.3807	7.8485 ± 5.9292	2.3577 ± 0.3961	1.1704 ± 0.253
45	tridecane	1227	8.4	1.28	C_13_H_28_	87	629505	184	not detected	not detected	not detected	1.4251 ± 0.2479	not detected
46	hexadecane	1299	9.45	2.53	C_16_H_34_	84	544763	226	7.5238 ± 5.2513	not detected	5.7390 ± 4.5180	1.5395 ± 0.0409	2.3853 ± 2.0450
47	3,8-dimethylundecane	1490	12.37	1.99	C_13_H_28_	80	17301303	184	2.9744 ± 0.4509	0.6414 ± 0.2790	1.6418 ± 0.4401	0.6497 ± 0.3586	2.0775 ± 0.2829
48	2,6,10,15-tetramethylheptadecane	1497	12.48	3.21	C_21_H_44_	80	54833486	296	not detected	not detected	not detected	0.2712 ± 0.0094	not detected
49	3-ethyl-3-methylheptane	1557	13.53	3.8	C_10_H_22_	78	17302011	142	not detected	not detected	not detected	0.3293 ± 0.1140	not detected
50	heptatriacontan-1-ol	1935	20.07	3.38	C_37_H_76_O	75	105794589	536	0.2469 ± 0.0489	0.0218 ± 0.0093	2.5082 ± 0.0568	0.0594 ± 0.0109	0.8715 ± 0.7472
51	undecane	1212	8.17	1.34	C_11_H_24_	86	1120214	156	8.3629 ± 1.7491	not detected	not detected	not detected	not detected
52	2,5,9-trimethyldecane	1227	8.4	1.35	C_13_H_28_	73	62108229	184	not detected	not detected	not detected	not detected	0.1544 ± 0.0072
	total alkanes								19.1080	1.4543	17.7375	6.6319	6.6591
	*others*												
53	2-(7-Heptadecynyloxy)tetrahydro-2H-pyran	1814	17.97	5.88	C_22_H_40_O_2_	69	56599509	336	0.2877 ± 0.0293	0.0747 ± 0.0176	0.2114 ± 0.1161	0.2749 ± 0.0608	0.3608 ± 0.1783
54	1-chlorooctadecane	1935	20.07	3.34	C_18_H_37_Cl	72	3386332	288	not detected	not detected	not detected	0.2630 ± 0.0122	0.0911 ± 0.0270
55	2,4-decadienal	1331	9.92	2.89	C_10_H_16_O	84	2363884	152	not detected	not detected	0.1276 ± 0.0761	not detected	0.1524 ± 0.0520
56	methyl 2,5-octadecadiynoate	2134	25.9	5.23	C_19_H_30_O_2_	72	57156919	290	not detected	not detected	0.4789 ± 0.0620	not detected	0.0159 ± 0.0075
57	bicyclohexyliden-2-one	2156	26.72	2.7	C_12_H_18_O	68	1011127	178	not detected	not detected	not detected	not detected	0.2567 ± 0.1567
58	methyl arachidonate	2225	29.17	4.19	C_21_H_34_O_2_	75	2566894	318	not detected	not detected	not detected	not detected	0.0108 ± 0.0025
59	vinyl stearate	1048	6.53	1.11	C_20_H_38_O_2_	59	111637	310	not detected	0.0900 ± 0.0214	not detected	not detected	not detected
60	6-(3,5-dimethylfuran-2-yl)-6-methylheptan-2-one	1941	20.18	5.81	C_14_H_22_O_2_	65	90165096	222	not detected	0.9870 ± 0.3374	not detected	not detected	not detected

### 3.2. Analyses of volatile compounds from various types of agarwood

**Figure 3.**
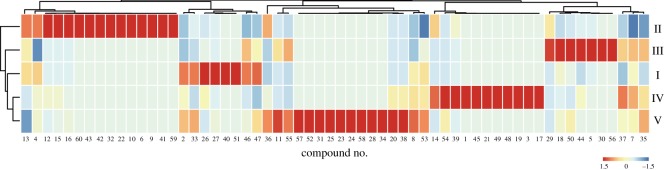
Distribution of identified compounds in the five types of agarwood.

Of the five agarwood types, Types IV and II contained the greatest number and concentration of distinct sesquiterpenes, respectively (table 1), and Types I and II contained greater monoterpene concentrations than Types IV and V. In addition, Type V had the greatest diterpene concentration, and Types I and III contained high levels of alkanes (table 1). A concentration heat map of volatile compounds was generated to further analyse the volatile profiles of the agarwood samples (figure 3). From this, Type II was clustered on a single branch of the cluster tree, which suggested that the volatile compounds of Type II were quite different from the others.

### 3.3. Structure and diversity of agarwood-associated fungal communities

**Figure 4.**
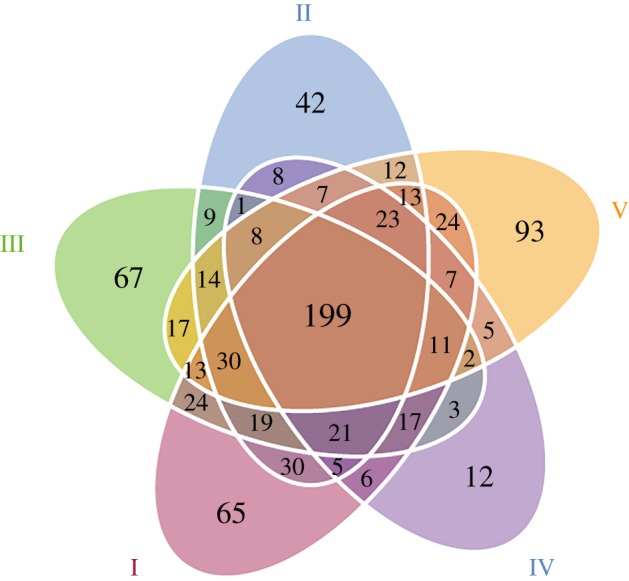
Distribution of fungal species among the five types of agarwood. Each circle represents one type of agarwood, and overlapping regions indicate common operational taxonomic units (OTUs); non-overlapping regions indicate type-specific OTUs.

**Figure 5.**
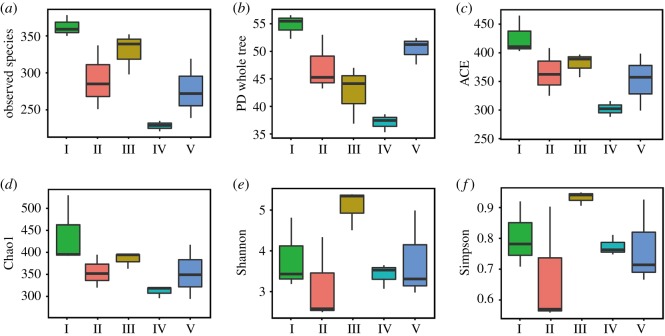
Richness and diversity of fungal communities associated with the five types of agarwood. The indices included observed species (*a*), PD whole tree (*b*), ACE (*c*), Chao 1 (*d*), Shannon (*e*) and Simpson (*f*) indices.

**Figure 6.**
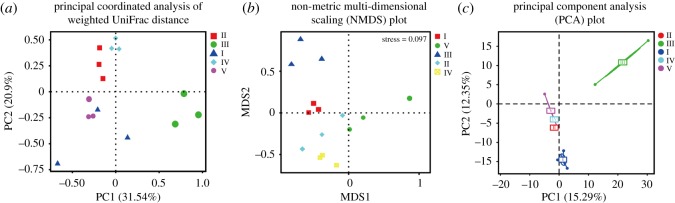
Evaluation of fungal communities by UniFrac analysis: (*a*) principal coordinated analysis of weighted UniFrac distance, which depicts the species composition structure of the five types of agarwood; (*b*) non-metric multi-dimensional scaling (NMDS) plot, which depicts the diversity of the fungal community by nonlinear structure; (*c*) principal component analysis (PCA) of the five types of agarwood.

Between 52 594 and 80 303 qualified reads were obtained from each of the five types of agarwood, and were attributed to six phyla, 25 classes, 63 orders, 115 families and 141 genera of fungi, with 221–378 OTUs identified with a similarity of 97% (table 2). The species diversity of Type IV was lower than that of the others (table 2), and the samples of each agarwood type possessed more common OTUs than specific ones (figure 4). Significantly more fungal species were observed in Type I than in Type IV (*p* < 0.05; figure 5*a*). Other community richness indices, including PD whole tree, ACE and Chao1, also indicated that the fungal community of Type I was richer than that of the other types and that the fungal community of Type IV possessed the lowest richness (figure 5*b*–*d*). However, the Shannon and Simpson indices identified Type III as possessing the greatest fungal diversity and Type II as possessing the lowest (figure 5*e*,*f*). UniFrac analysis, including PCoA, NMDS and PCA, indicated that Types III and I were distinct from Types II and IV (figure 6*a*–*c*).

**Table 2.** Diversity of fungal species in the five types of agarwood.

type	no.	phylum	class	order	family	genus	OTUs
I	1	5	20	41	71	64	378
	2	5	19	43	77	67	350
	3	4	16	40	68	61	359
II	1	4	18	43	73	66	337
	2	3	15	39	63	56	251
	3	4	17	37	66	57	285
III	1	2	12	31	47	44	298
	2	3	15	35	55	54	352
	3	4	15	39	62	60	339
IV	1	3	14	33	48	43	235
	2	3	16	35	50	41	229
	3	2	12	31	49	40	221
V	1	3	14	33	61	54	239
	2	3	18	40	68	59	272
	3	5	18	41	62	59	319

### 3.4. Distribution of fungi among agarwood types

**Figure 7.**
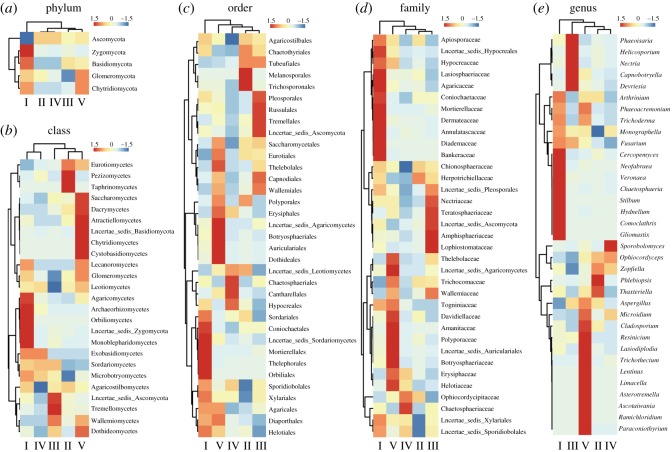
Distribution of fungal taxa in the five types of agarwood. Heat maps are based on the distribution of fungal phyla (*a*), classes (*b*), orders (*c*), families (*d*) and genera (*e*).

A heat map was generated to further analyse the taxonomic distribution of fungi among the different types of agarwood (figure 7). Among the five detected fungal phyla, the Ascomycota was the most dominant in all the agarwood samples, and Basidiomycota and Zygomycota were more abundant in Type I than in the other types of agarwood (figure 7*a*). Among the 25 detected fungal classes, the Agaricomycetes, Archaeorhizomycets, Orbiliomycetes and Monoblepharidomycetes were most abundant in Type I, whereas the Tremellomycetes was most abundant in Type III, the Pezizomycetes and Taphrinomycetes were most abundant in Type II, and the Saccharomycetes, Dacrymycetes, Atractiellomycetes, Chytridiomycetes and Cystobasidiomycetes were most abundant in Type V. By contrast, no fungal classes were particularly abundant in Type IV (figure 7*b*). Among the 10 most abundant fungal classes, the Sordariomycetes was dominant in Types I, III and IV, whereas the Dothideomycetes and Eurotiomycetes were dominant in Types II and V, respectively. The fungal orders and families of Type I were different to those of Types II, III, IV and V (figure 7*c*,*d*), and the dominant fungal orders and families of the five agarwood types were different (figure 7*b*,*c*). The most dominant genera of Types V, I, III and II/IV were *Lasiodiplodia*, *Hydnellum*, *Phaeoisaria* and *Ophiocordyceps*, respectively (figure 7*e*).

### 3.5. Correlation between agarwood chemistry and fungal diversity

The correlation between the chemical and fungal components of the agarwood was analysed to further examine the correlation between fungal species and agarwood composition. Based on the presence of different volatiles and fungal genera, correlation analysis revealed that the Zygomycota was associated with the distribution of aromatic compounds (*r* = 0.661**, *p* < 0.01; table 3). Correlations between the 30 most abundant fungal genera and levels of 60 compounds were analysed (table 4 and electronic supplementary material, table S1). Analysis of the 25 sesquiterpenes revealed that the presence of *Paraconiothyrium* and *Cladosporium* species was significantly correlated with compounds 11, 20 and 24; whereas the presence of *Limacella* and *Trichothecium* species was associated with compounds 24 and 25; the presence of *Resinicium* species was associated with compounds 23–25; and the presence of *Lasiodiplodia* species was associated with compound 23 (*r* > 0.8, *p* < 0.01; table 4 and table S1). Meanwhile, further investigation of the seven identified monoterpenes revealed that the presence of *Cercopemyces* and *Neofabraea* species was associated with compound 26; the presence of *Hydnellum*, *Monographella* and *Veronaea* species was correlated with compound 27; the presence of *Limacella*, *Lasiodiplodia*, *Ascotaiwania*, *Asterotremella* and *Ramichloridium* species was correlated with compound 28; the presence of *Helicosporium* and *Phlebiopsis* species was associated with compounds 29 and 32, respectively; the presence of both *Phaeoisaria* and *Capnobotryella* species was correlated with compound 30; and the presence of *Lentinus* and *Resinicium* species was associated with compound 31 (*r* > 0.8, *p* < 0.01; table 4 and table S1). In regard to diterpene distribution, six fungal genera were found to be correlated with compound 34, including *Limacella*, *Lasiodiplodia*, *Lentinus*, *Resinicium*, *Trichothecium* and *Ramichloridium*, whereas *Phaeoacremonium* was associated with compound 33 (*r* > 0.8, *p* < 0.01; table 4 and table S1). Of the nine aromatics, compounds 38, 39, 40, and 42 were correlated with the presence of *Microidium*, *Sporobolomyces*, *Gliomastix* and *Veronaea*, and *Thaxteriella,* respectively (*r* > 0.8, *p* < 0.01; table 4 and table S1). Among the nine alkanes, compound 52 was associated with the presence of *Paraconiothyrium*, *Ascotaiwannia* and *Phaeoacremonium*, whereas compounds 44, 49 and 51 were associated with *Devriesin*, *Sporobolomyces* and *Comoclathris*, respectively (*r* > 0.8, *p* < 0.01; table 4 and table S1).

**Table 3.** Correlation of volatiles and fungal phyla among the five types of agarwood.

	sesquiterpenes	monoterpenes	diterpenes	aromatics	alkanes	Zygomycota	Chytridiomycota	Ascomycota	Glomeromycota	Basidiomycota
sesquiterpenes	1.000	0.161**	0.099**	−0.010	−0.727**	−0.167**	−0.059**	0.289**	0.324**	−0.288**
monoterpenes	0.161**	1.000	−0.326**	−0.176**	0.112**	0.191**	−0.058**	−0.335**	−0.058**	0.334**
diterpenes	0.099**	−0.326**	1.000	−0.193**	−0.199**	−0.137**	0.354**	0.070**	0.310**	−0.070**
aromatics	−0.010	−0.176**	−0.193**	1.000	−0.016*	0.661**	−0.149**	−0.288**	0.296**	0.279**
alkanes	−0.727**	0.112**	−0.199**	−0.016*	1.000	0.189**	0.300**	−0.481**	−0.167**	0.480**
Zygomycota	−0.167**	0.191**	−0.137**	0.661**	0.189**	1.000	0.094**	−0.286**	0.145**	0.272**
Chytridiomycota	−0.059**	−0.058**	0.354**	−0.149**	0.300**	0.094**	1.000	−0.204**	0.607**	0.200**
Ascomycota	0.289**	−0.335**	0.070**	−0.288**	−0.481**	−0.286**	−0.204**	1.000	−0.330**	−1.000**
Glomeromycota	0.324**	−0.058**	0.310**	0.296**	−0.167**	0.145**	0.607**	−0.330**	1.000	0.327**
Basidiomycota	−0.288**	0.334**	−0.070**	0.279**	0.480**	0.272**	0.200**	−1.000**	0.327**	1.000

**p < 0.01; *p < 0.05; italic labelled number, correlation coefficient *r*-value greater than 0.6.

**Table 4.** Correlation between volatile compounds and fungal genera.

compound		fungal genus	*r*-value	compound		fungal genus	*r*-value
sesquiterpenes	compound 5	*Helicosporium*	0.85**	monoterpenes	compound 28	*Limacella*	0.81**
		*Nectria*	0.91**			*Lasiodiplodia*	0.98**
	compound 10	*Thaxteriella*	0.93**			*Ascotaiwania*	0.91*
	compound 11	*Paraconiothyrium*	0.89**			*Asterotremella*	0.91**
		*Cladosporium*	0.87**			*Ramichloridium*	0.90**
	compound 12	*Phlebiopsis*	0.89**		compound 29	*Helicosporium*	0.81**
	compound 16	*Phlebiopsis*	1.00**		compound 30	*Phaeoisaria*	0.86**
	compound 18	*Helicosporium*	0.94**			*Capnobotryella*	0.87**
	compound 20	*Paraconiothyrium*	0.95**		compound 31	*Lentinus*	0.98**
		*Cladosporium*	0.93**			*Resinicium*	0.92**
		*Phaeoacremonium*	0.83**		compound 32	*Phlebiopsis*	0.88**
	compound 22	*Phlebiopsis*	0.91**	diterpenes	compound 33	*Phaeoacremonium*	0.91**
	compound 23	*Lasiodiplodia*	0.98**		compound 34	*Limacella*	0.93**
		*Lentinus*	0.87**			*Lasiodiplodia*	0.83**
		*Resinicium*	0.89**			*Lentinus*	0.85**
	compound 24	*Paraconiothyrium*	0.85**			*Resinicium*	0.98**
		*Limacella*	0.81**			*Trichothecium*	0.87**
		*Cladosporium*	0.85**			*Ramichloridium*	0.83**
		*Resinicium*	0.87**	aromatics	compound 38	*Microidium*	0.94**
		*Trichothecium*	0.97**		compound 39	*Sporobolomyces*	0.95**
	compound 25	*Limacella*	0.99**		compound 40	*Gliomastix*	0.91**
		*Ascotaiwania*	0.83**			*Veronaea*	1.00**
		*Asterotremella*	0.83**		compound 43	*Thaxteriella*	0.95**
		*Resinicium*	0.87**	alkanes	compound 44	*Devriesia*	0.86**
		*Trichothecium*	0.95**		compound 49	*Sporobolomyces*	0.99**
		*Ramichloridium*	0.88**		compound 51	*Comoclathris*	0.93**
monoterpenes	compound 26	*Cercopemyces*	0.87**		compound 52	*Paraconiothyrium*	0.99**
		*Neofabraea*	0.91**			*Cladosporium*	0.98**
	compound 27	*Hydnellum*	0.85**			*phaeoacremonium*	0.89**
		*Veronaea*	0.92**				
		*Monographella*	0.83**				

*p < 0.05; **p < 0.01.

Together, these analyses indicate that the presence of *Lasiodiplodia*, *Cladosporium*, *Resinicium*, *Hydnellum* and *Monographella* is highly correlated with volatiles in eight common fungal genera that were present in all agarwood samples. Although *Fusarium*, *Trichoderma* and *Aspergillus* were not highly correlated with the volatiles (*r* < 0.8), the genera have been reported to play roles in agarwood formation [42–45].

## 4. Discussion

Agarwood is a typical example of the raw materials for perfume and oriental medicine, with the most potent compounds being sesquiterpene, monoterpene and aromatic compounds [3]. Natural agarwood can be divided into different types, owing to the different formation locations and incenses. The present study is the first work to investigate the chemical characteristics of agarwood types formed at different wound locations. Using GC × GC-HR-TOF-MS, the present study determined that differences in the type and number of compounds in the different types of agarwood were significant and that GC × GC-HR-TOF-MS can be used as an effective tool for the separation and identification of individual compounds from complex volatile oils.

Previous studies have reported that certain fungal strains obtained from *Aquilaria* can produce volatile compounds similar to those found in the essential oil of agarwood [46]. UniFrac analysis indicated that the crosscutting (including Types I and III) and rip cutting (Type IV) wounding methods affected the surrounding microbial communities, thereby indicating that regional wound differences may contribute to the variation observed in agarwood (figure 6). Distribution analysis revealed that the fungal communities of agarwood types vary significantly at sub-order levels of classification. Moreover, functional fungal genera were further investigated through heat map analysis of the 35 most abundant fungal genera (figure 7). Although the abundance of each fungal genus differed among the agarwood types, eight common genera were found in all the agarwood samples, including *Fusarium*, *Lasiodiplodia*, *Cladosporium*, *Trichoderma*, *Aspergillus*, *Resinicium*, *Hydnellum* and *Monographella*, which suggests that these eight fungal genera play key roles in agarwood formation. However, only *Lasiodiplodia* [21], *Fusarium* [47] and *Trichoderma* [48] fungi from *A. sinensis* have been shown to induce agarwood formation.

The results of the present study demonstrate that the chemical properties of *A. sinensis*-derived agarwood are strongly associated with fungal diversity. For example, *Lasiodiplodia* and *Cladosporium* were highly correlated with certain sesquiterpene, monoterpene and diterpene compounds (table 4 and table S1), whereas the abundances of *Hydnellum* and *Monographella* were correlated with certain monoterpene compounds. However, even though the presence of these five genera were highly correlated with the biosynthesis of agarwood volatiles, only *Lasiodiplodia* sp. has been reported that could promote agarwood formation. *Lasiodiplodia theobromae* isolated from *A. sinensis* has been reported to produce jasmonic acids that induce significant increases in sesquiterpene contents and chromate stimulation [49,50], which could, thereby, promote agarwood formation [21]. In the present study, the presence of *Fusarium*, *Trichoderma* and *Aspergillus* was also correlated with volatile contents (table S1), and other studies have also reported that the genera play roles in agarwood formation [42–45]. Indeed, *Fusarium* has been reported to induce the formation of agarwood in *A. malaccensis* and is capable of producing certain agarwood compounds, such as tridecanoic acid, α-santalol and spathulenol [43]. *Fusarium* has also been reported to produce specific secondary metabolites, such as pyrone derivatives [51]. Meanwhile, *Trichoderma* has been reported to promote the production of both sesquiterpenes and chromone derivatives in *A. malaccensis* cell suspension cultures [44], and *Aspergillus*, one of the most predominant fungal genera in agarwood, has been shown to induce the biosynthesis of mycotoxins, including aflatoxin B1 (AFB1) and ochratoxin A (OTA) [45]. Together, the results of the present study demonstrate that the chemical properties of *A. sinensis* are strongly associated with fungal diversity. However, it remains unclear whether the volatile constituents of agarwood are synthesized by the fungi or by the host trees. Accordingly, future research should focus more on the underlying mechanisms of agarwood formation, such as the role of functional genes in the interaction between *Aquilaria*, fungi and agarwood compounds.

## 5. Conclusion

The aim of the present study was to investigate the relationship between the chemistry and fungal associates of agarwood formed at different spatial locations. The findings presented here reveal that the location of agarwood formation significantly affects the chemical and fungal constituents of agarwood in *A. sinensis*. The occurrence of terpenoids, such as sesquiterpenes, monoterpenes and diterpenes, was closely related to fungal diversity, which is a primary determinant of agarwood properties. In agreement with previous studies that have reported that the volatile oil of agarwood can inhibit fungal growth, the results of the present study indicate that the volatile compounds of agarwood directly affect fungal diversity, which could further influence agarwood formation.

## Supplementary Material

Figure S1

Reviewer comments

## Supplementary Material

Table S1

## Data accessibility

Raw reads of the high-throughput sequencing data: Sequence Read Achieve (SRA) database (BioProject number: PRJNA509099).
