# Supplementary material for: Agarwood wound locations provide insight into the association between fungal diversity and volatile compounds in Aquilaria sinensis
Source: R Soc Open Sci. 2019 Jul 3;6(7):190211. doi: 10.1098/rsos.190211 (PMC6689645; doi:10.1098/rsos.190211)
Supplement: Figure S1 [file rsos190211supp1.docx]

**Agarwood wound locations provide insight into the association between fungal diversity and volatile compounds in *Aquilaria sinensis***

Juan Liu^†^, Xiang Zhang^†^, Jian Yang, Junhui Zhou, Yuan Yuan^*^, Chao Jiang, Xiulian Chi, Luqi Huang ^*^

Y.Y. (y_yuan0732@163.com)

L.H. (huangluqi01@126.com)

**List of Supplemental Figure**

**Supplemental Figure S1.** Structures of α-eudesmol and α-copaen-11-ol, the spectra of a sample peak, and in the NIST library, respectively.


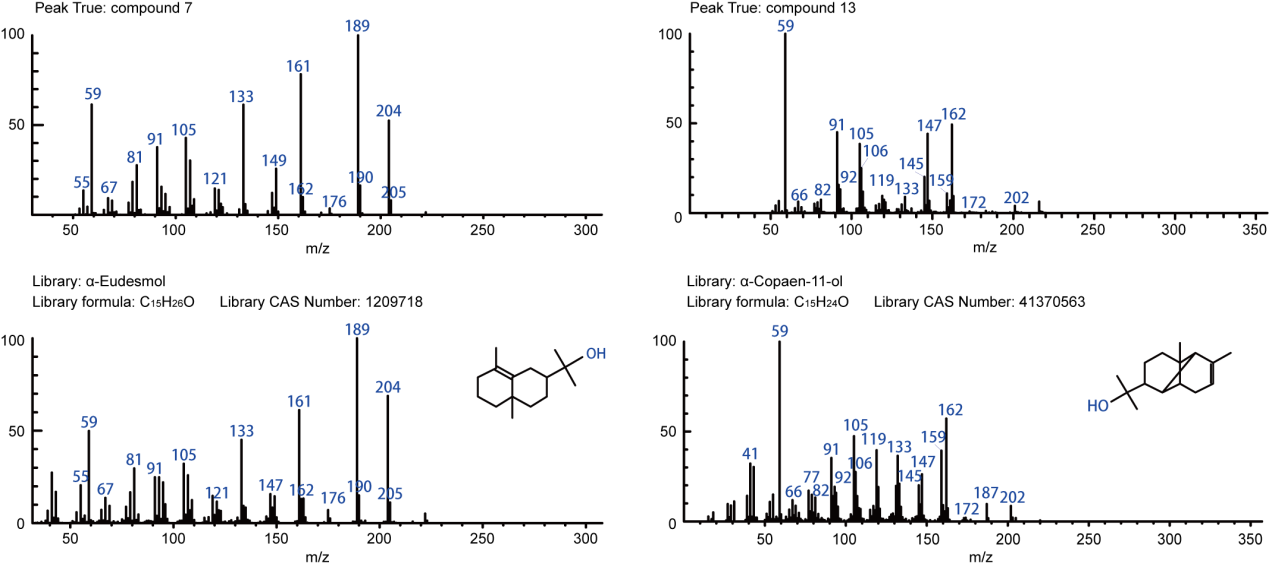


Supplemental Figure S1. Structures of α-eudesmol and α-copaen-11-ol, the spectra of a sample peak, and in the NIST library, respectively.
