# Supplementary material for: Agarwood wound locations provide insight into the association between fungal diversity and volatile compounds in Aquilaria sinensis
Source: R Soc Open Sci. 2019 Jul 3;6(7):190211. doi: 10.1098/rsos.190211 (PMC6689645; doi:10.1098/rsos.190211)
Supplement: Table S1 [file rsos190211supp2.docx]

**Agarwood wound locations provide insight into the association between fungal diversity and volatile compounds in *Aquilaria sinensis***

Juan Liu^†^, Xiang Zhang^†^, Jian Yang, Junhui Zhou, Yuan Yuan^*^, Chao Jiang, Xiulian Chi, Luqi Huang ^*^

Y.Y. (y_yuan0732@163.com)

L.H. (huangluqi01@126.com)

**List of Supplemental Table**

**Supplemental Table S1.** Correlation between the volatile compounds and fungal communities of agarwood. *Par*, Paraconiothyrium; *Sti*, Stilbum; *Lim*, Limacella; *Zop*, Zopfiella; *Art*, Arthrinium; *Dev*, Devriesia; *Fus*, Fusarium; *Las*, Lasiodiplodia; *Asc*, Ascotaiwania; *Oph*, Ophiocordyceps; *Cla*, Cladosporium; *Cer*, Cercopemyces; *Tri*, Trichoderma; *Phl*, Phlebiopsis; *Ast*, Asterotremella; *Hel*, Helicosporium; *Mic*, Microidium; *Gli*, Gliomastix; *Asp*, Aspergillus; *Com*, Comoclathris; *Spo*, Sporobolomyces; *Len*, Lentinus; *Pha*, Phaeoisaria; *Res*, Resinicium; *Hyd*, Hydnellum; *Cap*, Capnobotryella; *Neo*, Neofabraea; *Tri*, Trichothecium; Ver, Veronaea; *Pha*, Phaeoacremonium; *Ram*, Ramichloridium; *Cha*, Chaetosphaeria; *Tha*, Thaxteriella; *Mon*, Monographella; *Nec*, Nectria. **. p<0.01; *. p<0.05; bold labeled number, correlation coefficent r-value > 0.8.

**Supplemental Table S1**. Correlation between the volatile compounds and fungal communities of agarwood. *Par*, Paraconiothyrium; *Sti*, Stilbum; *Lim*, Limacella; *Zop*, Zopfiella; *Art*, Arthrinium; *Dev*, Devriesia; *Fus*, Fusarium; *Las*, Lasiodiplodia; *Asc*, Ascotaiwania; *Oph*, Ophiocordyceps; *Cla*, Cladosporium; *Cer*, Cercopemyces; *Tri*, Trichoderma; *Phl*, Phlebiopsis; *Ast*, Asterotremella; *Hel*, Helicosporium; *Mic*, Microidium; *Gli*, Gliomastix; *Asp*, Aspergillus; *Com*, Comoclathris; *Spo*, Sporobolomyces; *Len*, Lentinus; *Pha*, Phaeoisaria; *Res*, Resinicium; *Hyd*, Hydnellum; *Cap*, Capnobotryella; *Neo*, Neofabraea; *Tri*, Trichothecium; Ver, Veronaea; *Pha*, Phaeoacremonium; *Ram*, Ramichloridium; *Cha*, Chaetosphaeria; *Tha*, Thaxteriella; *Mon*, Monographella; *Nec*, Nectria.

|  | ***Par*** | ***Sti*** | ***Lim*** | ***Zop*** | ***Art*** | ***Dev*** | ***Fus*** | ***Las*** | ***Asc*** | ***Oph*** | ***Cla*** | ***Cer*** | ***Tri*** | ***Phl*** | ***Ast*** | ***Hel*** | ***Mic*** | ***Gli*** |
| --- | --- | --- | --- | --- | --- | --- | --- | --- | --- | --- | --- | --- | --- | --- | --- | --- | --- | --- |
| Compound 1 | -0.10 | -0.12 | -0.14 | 0.29 | -0.27 | -0.16 | -0.26 | -0.14 | -0.10 | 0.36 | -0.13 | -0.10 | -0.17 | -0.10 | -0.10 | -0.12 | -0.19 | -0.10 |
| Compound 2 | 0.47^*^ | 0.05 | 0.49^*^ | -0.13 | 0.33 | -0.27 | 0.16 | 0.15 | 0.30 | -0.02 | 0.46^*^ | -0.18 | 0.43 | -0.18 | 0.30 | -0.22 | -0.02 | 0.52^*^ |
| Compound 3 | -0.11 | -0.14 | -0.15 | -0.03 | -0.31 | -0.18 | -0.36 | -0.17 | -0.11 | 0.54^*^ | -0.12 | -0.12 | -0.20 | -0.12 | -0.11 | -0.14 | 0.25 | -0.11 |
| Compound 4 | -0.27 | 0.12 | 0.28 | 0.21 | 0.55^*^ | -0.39 | -0.24 | 0.20 | 0.43 | 0.39 | -0.21 | 0.10 | -0.02 | 0.21 | 0.43 | -0.32 | 0.00 | 0.14 |
| Compound 5 | -0.10 | -0.14 | -0.12 | -0.32 | -0.29 | 0.30 | 0.53^*^ | -0.12 | -0.08 | -0.26 | -0.14 | -0.12 | -0.19 | -0.12 | -0.08 | **0.85^**^** | -0.17 | -0.12 |
| Compound 6 | -0.13 | -0.16 | -0.18 | 0.65^**^ | 0.15 | -0.20 | -0.30 | -0.19 | -0.13 | 0.11 | -0.08 | -0.12 | -0.21 | 0.52^*^ | -0.13 | -0.15 | -0.23 | -0.13 |
| Compound 7 | 0.06 | 0.03 | 0.35 | -0.40 | 0.12 | 0.20 | 0.13 | 0.07 | 0.35 | -0.14 | 0.04 | -0.19 | -0.11 | -0.13 | 0.35 | 0.25 | -0.22 | 0.10 |
| Compound 8 | -0.22 | 0.07 | 0.32 | -0.26 | 0.24 | -0.33 | -0.10 | 0.78^**^ | 0.46^*^ | -0.13 | -0.23 | -0.05 | 0.46^*^ | -0.22 | 0.46^*^ | -0.27 | 0.67^**^ | 0.10 |
| Compound 9 | -0.11 | -0.13 | -0.14 | 0.66^**^ | 0.05 | -0.16 | -0.26 | -0.15 | -0.10 | 0.00 | -0.10 | -0.10 | -0.17 | 0.16 | -0.10 | -0.13 | -0.21 | -0.10 |
| Compound 10 | -0.08 | -0.09 | -0.11 | 0.62^**^ | -0.19 | -0.12 | -0.20 | -0.11 | -0.08 | 0.66^**^ | -0.10 | -0.07 | -0.12 | -0.06 | -0.08 | -0.10 | -0.16 | -0.08 |
| Compound 11 | **0.89^**^** | -0.12 | 0.32 | -0.08 | -0.27 | -0.03 | 0.32 | -0.13 | -0.09 | 0.01 | **0.87^**^** | -0.10 | -0.04 | -0.10 | -0.09 | 0.36 | 0.02 | -0.10 |
| Compound 12 | -0.13 | -0.16 | -0.18 | 0.32 | 0.28 | -0.20 | -0.29 | -0.19 | -0.13 | 0.03 | -0.02 | -0.12 | -0.21 | **0.89^**^** | -0.13 | -0.13 | -0.19 | -0.13 |
| Compound 13 | -0.30 | 0.25 | -0.34 | 0.54^*^ | -0.08 | -0.01 | 0.11 | -0.37 | -0.21 | 0.32 | -0.32 | 0.19 | -0.24 | -0.16 | -0.21 | 0.15 | -0.49^*^ | -0.04 |
| Compound 14 | -0.19 | -0.23 | -0.26 | 0.47^*^ | -0.17 | -0.30 | -0.49^*^ | -0.28 | -0.19 | .547^*^ | -0.16 | -0.19 | -0.32 | 0.35 | -0.19 | -0.22 | -0.17 | -0.19 |
| Compound 15 | -0.12 | -0.19 | -0.15 | .651^**^ | 0.14 | -0.24 | -0.36 | -0.19 | -0.11 | 0.27 | -0.05 | -0.15 | -0.24 | 0.63^**^ | -0.11 | -0.18 | -0.24 | -0.16 |
| Compound 16 | 0.00 | -0.10 | -0.08 | -0.03 | 0.37 | -0.13 | -0.14 | -0.11 | -0.08 | -0.15 | 0.13 | -0.08 | -0.11 | **1.00^**^** | -0.08 | -0.07 | -0.07 | -0.09 |
| Compound 17 | -0.11 | -0.14 | -0.15 | 0.05 | -0.31 | -0.18 | -0.34 | -0.16 | -0.11 | .508^*^ | -0.13 | -0.11 | -0.20 | -0.11 | -0.11 | -0.14 | 0.15 | -0.11 |
| Compound 18 | 0.03 | -0.02 | -0.11 | -0.29 | -0.26 | 0.29 | 0.68^**^ | -0.11 | -0.13 | -0.28 | 0.00 | 0.09 | -0.15 | -0.16 | -0.13 | **0.94^**^** | -0.13 | -0.14 |
| Compound 19 | -0.08 | -0.10 | -0.11 | -0.19 | -0.22 | -0.13 | -0.28 | -0.12 | -0.08 | .441^*^ | -0.08 | -0.08 | -0.14 | -0.08 | -0.08 | -0.10 | 0.38 | -0.08 |
| Compound 20 | **0.95^**^** | -0.13 | 0.35 | 0.06 | -0.28 | -0.17 | -0.04 | -0.05 | -0.09 | 0.17 | **0.93^**^** | -0.11 | 0.03 | -0.11 | -0.09 | -0.13 | 0.14 | -0.10 |
| Compound 21 | -0.12 | -0.15 | -0.17 | 0.02 | -0.33 | -0.20 | -0.38 | -0.18 | -0.12 | 0.56^*^ | -0.14 | -0.12 | -0.21 | -0.12 | -0.12 | -0.15 | 0.19 | -0.12 |
| Compound 22 | -0.11 | -0.13 | -0.15 | 0.27 | 0.29 | -0.17 | -0.22 | -0.16 | -0.11 | 0.08 | 0.00 | -0.10 | -0.17 | **0.91^**^** | -0.11 | -0.10 | -0.16 | -0.11 |
| Compound 23 | 0.10 | -0.14 | 0.75^**^ | -0.28 | 0.32 | -0.16 | -0.17 | **0.98^**^** | 0.76^**^ | -0.25 | 0.10 | -0.12 | 0.26 | -0.11 | 0.76^**^ | -0.15 | 0.74^**^ | -0.12 |
| Compound 24 | **0.85^**^** | -0.13 | **0.81^**^** | -0.11 | 0.11 | -0.16 | -0.09 | 0.40 | 0.45^*^ | -0.03 | **0.85^**^** | -0.11 | 0.05 | -0.11 | 0.45^*^ | -0.14 | 0.33 | -0.11 |
| Compound 25 | 0.50^*^ | -0.12 | **0.99^**^** | -0.17 | 0.40 | -0.13 | -0.18 | 0.64^**^ | **0.83^**^** | -0.11 | 0.50^*^ | -0.10 | -0.04 | -0.09 | **0.83^**^** | -0.13 | 0.30 | -0.10 |
| Compound 26 | -0.14 | 0.33 | -0.20 | -0.08 | 0.10 | -0.09 | 0.11 | -0.21 | -0.14 | -0.06 | -0.13 | **0.87^**^** | -0.22 | -0.15 | -0.14 | -0.12 | -0.07 | -0.14 |
| Compound 27 | -0.10 | 0.70^**^ | -0.13 | -0.31 | 0.44 | -0.17 | 0.43 | -0.11 | -0.10 | -0.27 | -0.10 | -0.09 | 0.60^**^ | -0.11 | -0.10 | -0.13 | -0.17 | 0.69^**^ |
| Compound 28 | -0.06 | -0.12 | **0.81^**^** | -0.27 | 0.46^*^ | -0.13 | -0.20 | **0.98^**^** | **0.91^*^** | -0.25 | -0.06 | -0.10 | 0.15 | -0.09 | **0.91^**^** | -0.13 | 0.60^**^ | -0.10 |
| Compound 29 | -0.17 | -0.22 | -0.25 | -0.04 | -0.35 | 0.57^*^ | 0.38 | -0.26 | -0.18 | -0.16 | -0.21 | -0.18 | -0.32 | -0.01 | -0.18 | **0.81^**^** | -0.36 | -0.18 |
| Compound 30 | -0.12 | -0.16 | -0.18 | -0.38 | -0.36 | 0.75^**^ | 0.41 | -0.19 | -0.13 | -0.34 | -0.17 | -0.14 | -0.24 | -0.14 | -0.13 | 0.75^**^ | -0.27 | -0.13 |
| Compound 31 | 0.61^**^ | -0.15 | 0.59^*^ | -0.20 | -0.02 | -0.19 | -0.06 | 0.66^**^ | 0.34 | -0.13 | 0.61^**^ | -0.13 | 0.33 | -0.13 | 0.34 | -0.16 | 0.73^**^ | -0.13 |
| Compound 32 | -0.10 | -0.12 | -0.14 | 0.23 | 0.35 | -0.16 | -0.21 | -0.15 | -0.10 | -0.19 | 0.00 | -0.09 | -0.16 | **0.88^**^** | -0.10 | -0.10 | -0.15 | -0.10 |
| Compound 33 | 0.77^**^ | 0.38 | 0.28 | -0.03 | 0.14 | -0.25 | 0.24 | -0.14 | -0.09 | -0.10 | 0.77^**^ | 0.39 | 0.10 | -0.12 | -0.09 | -0.20 | -0.07 | 0.06 |
| Compound 34 | 0.44^*^ | -0.15 | **0.93^**^** | -0.23 | 0.32 | -0.17 | -0.17 | **0.83^**^** | 0.78^**^ | -0.17 | 0.45 | -0.13 | 0.14 | -0.12 | 0.78^**^ | -0.16 | 0.57^*^ | -0.13 |
| Compound 35 | 0.29 | -0.19 | 0.26 | -0.25 | -0.31 | 0.22 | 0.12 | 0.19 | 0.14 | -0.09 | 0.23 | -0.21 | -0.01 | -0.29 | 0.14 | 0.28 | 0.11 | -0.12 |
| Compound 36 | 0.62^**^ | -0.28 | 0.29 | 0.26 | -0.06 | -0.37 | -0.34 | 0.18 | 0.00 | 0.26 | 0.67^**^ | -0.23 | 0.03 | 0.37 | 0.00 | -0.28 | 0.44^*^ | -0.24 |
| Compound 37 | 0.18 | -0.22 | 0.02 | 0.04 | -0.39 | 0.13 | 0.06 | -0.13 | -0.07 | 0.18 | 0.14 | -0.18 | -0.23 | -0.17 | -0.07 | 0.37 | -0.11 | -0.18 |
| Compound 38 | -0.10 | -0.13 | 0.04 | -0.28 | -0.16 | -0.17 | -0.12 | 0.64^**^ | 0.09 | -0.06 | -0.11 | -0.11 | 0.42 | -0.11 | 0.09 | -0.14 | **0.94^**^** | -0.11 |
| Compound 39 | -0.13 | -0.15 | -0.18 | 0.17 | -0.29 | -0.21 | -0.39 | -0.19 | -0.13 | 0.54^*^ | -0.14 | -0.13 | -0.22 | -0.04 | -0.13 | -0.16 | 0.03 | -0.13 |
| Compound 40 | -0.10 | 0.40 | -0.14 | -0.27 | 0.45^*^ | -0.15 | 0.51^*^ | -0.14 | -0.10 | -0.24 | -0.10 | -0.09 | 0.74^**^ | -0.10 | -0.10 | -0.11 | -0.19 | **0.91^**^** |
| Compound 41 | -0.13 | -0.16 | -0.18 | 0.64^**^ | 0.11 | -0.20 | -0.30 | -0.19 | -0.13 | 0.32 | -0.07 | -0.12 | -0.21 | 0.56^*^ | -0.13 | -0.15 | -0.23 | -0.13 |
| Compound 42 | -0.14 | -0.16 | -0.18 | 0.68^**^ | 0.08 | -0.20 | -0.30 | -0.19 | -0.13 | 0.33 | -0.08 | -0.12 | -0.21 | 0.50^*^ | -0.13 | -0.15 | -0.24 | -0.13 |
| Compound 43 | -0.09 | -0.10 | -0.12 | 0.59^*^ | -0.13 | -0.13 | -0.21 | -0.13 | -0.09 | 0.64^**^ | -0.08 | -0.08 | -0.13 | 0.09 | -0.09 | -0.11 | -0.16 | -0.09 |
| Compound 44 | -0.14 | -0.22 | -0.07 | -0.23 | -0.33 | **0.86^**^** | 0.07 | -0.08 | 0.00 | -0.15 | -0.19 | -0.18 | -0.27 | -0.15 | 0.00 | 0.45^*^ | -0.19 | -0.18 |
| Compound 45 | -0.08 | -0.10 | -0.11 | -0.18 | -0.22 | -0.13 | -0.28 | -0.12 | -0.08 | 0.45^*^ | -0.08 | -0.08 | -0.14 | -0.08 | -0.08 | -0.10 | 0.37 | -0.08 |
| Compound 46 | 0.06 | 0.14 | -0.17 | -0.31 | -0.18 | 0.30 | 0.51^*^ | -0.15 | -0.21 | -0.28 | 0.04 | 0.58^*^ | -0.16 | -0.21 | -0.21 | 0.51^*^ | 0.01 | -0.20 |
| Compound 47 | 0.23 | 0.34 | 0.19 | -0.52^*^ | 0.43 | -0.07 | 0.70^**^ | 0.14 | 0.09 | -0.60^**^ | 0.23 | 0.45^*^ | 0.53^*^ | -0.20 | 0.09 | 0.10 | 0.04 | 0.49^*^ |
| Compound 48 | -0.11 | -0.13 | -0.15 | 0.21 | -0.30 | -0.18 | -0.31 | -0.16 | -0.11 | 0.44^*^ | -0.14 | -0.11 | -0.19 | -0.11 | -0.11 | -0.13 | -0.07 | -0.11 |
| Compound 49 | -0.13 | -0.16 | -0.18 | 0.11 | -0.35 | -0.21 | -0.40 | -0.19 | -0.13 | 0.58^*^ | -0.15 | -0.13 | -0.22 | -0.13 | -0.13 | -0.16 | 0.09 | -0.13 |
| Compound 50 | 0.07 | -0.08 | -0.06 | -0.25 | -0.25 | 0.19 | 0.09 | -0.03 | -0.11 | -0.21 | 0.04 | -0.07 | -0.02 | -0.11 | -0.11 | 0.10 | 0.00 | -0.08 |
| Compound 51 | -0.13 | 0.51^*^ | -0.18 | -0.15 | 0.48^*^ | -0.20 | 0.52^*^ | -0.19 | -0.13 | -0.32 | -0.12 | 0.66^**^ | 0.40 | -0.13 | -0.13 | -0.15 | -0.26 | 0.53^*^ |
| Compound 52 | **0.99^**^** | -0.10 | 0.47^*^ | -0.01 | -0.16 | -0.13 | 0.01 | 0.02 | 0.01 | 0.08 | **0.98^**^** | -0.09 | 0.05 | -0.08 | 0.01 | -0.11 | 0.16 | -0.08 |
| Compound 53 | 0.52^*^ | 0.50^*^ | 0.29 | -0.11 | -0.08 | 0.05 | -0.14 | -0.07 | 0.05 | 0.02 | 0.49^*^ | -0.18 | -0.21 | -0.19 | 0.05 | -0.15 | -0.12 | -0.22 |
| Compound 54 | 0.06 | -0.14 | 0.08 | -0.22 | -0.19 | -0.18 | -0.32 | 0.03 | 0.05 | 0.43 | 0.06 | -0.12 | -0.11 | -0.11 | 0.05 | -0.14 | 0.50^*^ | -0.11 |
| Compound 55 | 0.54^*^ | -0.23 | 0.43 | -0.39 | -0.25 | 0.33 | 0.09 | 0.34 | 0.20 | -0.30 | 0.51^*^ | -0.20 | 0.04 | -0.20 | 0.20 | 0.17 | 0.32 | -0.20 |
| Compound 56 | -0.09 | -0.11 | -0.11 | -0.27 | -0.24 | 0.42 | 0.08 | -0.11 | -0.07 | -0.24 | -0.12 | -0.10 | -0.16 | -0.10 | -0.07 | 0.21 | -0.17 | -0.09 |
| Compound 57 | 0.16 | -0.14 | 0.43 | -0.26 | 0.05 | -0.17 | -0.08 | **0.85^**^** | 0.38 | -0.22 | 0.15 | -0.12 | 0.43 | -0.11 | 0.38 | -0.14 | **0.87^**^** | -0.12 |
| Compound 58 | 0.35 | -0.16 | 0.68^**^ | -0.26 | 0.15 | -0.19 | -0.12 | **0.87^**^** | 0.56^*^ | -0.20 | 0.35 | -0.13 | 0.33 | -0.13 | 0.56^*^ | -0.16 | 0.79^**^ | -0.13 |
| Compound 59 | -0.13 | -0.16 | -0.18 | 0.63^**^ | 0.11 | -0.20 | -0.29 | -0.19 | -0.13 | 0.32 | -0.07 | -0.12 | -0.20 | 0.57^*^ | -0.13 | -0.14 | -0.23 | -0.13 |
| Compound 60 | -0.09 | -0.11 | -0.12 | 0.70^**^ | -0.18 | -0.14 | -0.23 | -0.13 | -0.09 | 0.63^**^ | -0.11 | -0.09 | -0.14 | -0.06 | -0.09 | -0.12 | -0.18 | -0.09 |

|  | ***Asp*** | ***Com*** | ***Spo*** | ***Len*** | ***Pha*** | ***Res*** | ***Hyd*** | ***Cap*** | ***Neo*** | ***Tri*** | ***Ver*** | ***Phm*** | ***Ram*** | ***Cha*** | ***Tha*** | ***Mon*** | ***Nec*** |
| --- | --- | --- | --- | --- | --- | --- | --- | --- | --- | --- | --- | --- | --- | --- | --- | --- | --- |
| Compound 1 | -0.24 | -0.16 | 0.61^**^ | -0.18 | -0.17 | -0.20 | -0.16 | -0.19 | -0.13 | -0.12 | -0.14 | -0.15 | -0.11 | -0.10 | -0.17 | -0.05 | -0.13 |
| Compound 2 | 0.22 | 0.28 | -0.05 | 0.28 | -0.30 | 0.45^*^ | 0.18 | -0.27 | -0.13 | 0.57^*^ | 0.50^*^ | 0.49^*^ | 0.35 | 0.05 | -0.09 | 0.40 | -0.24 |
| Compound 3 | -0.15 | -0.18 | 0.64^**^ | -0.21 | -0.19 | -0.23 | -0.18 | -0.22 | -0.15 | -0.16 | -0.16 | -0.18 | -0.13 | -0.11 | -0.19 | 0.02 | -0.15 |
| Compound 4 | -0.07 | 0.18 | -0.07 | -0.13 | -0.45^*^ | 0.01 | 0.17 | -0.40 | 0.12 | 0.07 | 0.18 | -0.20 | 0.40 | 0.10 | 0.59^*^ | -0.15 | -0.34 |
| Compound 5 | 0.30 | -0.19 | -0.17 | -0.16 | 0.75^**^ | -0.13 | -0.19 | 0.69^**^ | -0.16 | -0.14 | -0.16 | -0.17 | -0.08 | -0.12 | -0.20 | -0.12 | **0.91^**^** |
| Compound 6 | 0.05 | -0.21 | -0.23 | -0.24 | -0.22 | -0.26 | -0.21 | -0.24 | -0.18 | -0.20 | -0.19 | -0.20 | -0.14 | -0.13 | 0.40 | -0.55^*^ | -0.17 |
| Compound 7 | 0.21 | -0.05 | 0.18 | -0.08 | 0.20 | 0.14 | 0.03 | 0.26 | -0.16 | 0.28 | 0.11 | 0.06 | 0.36 | 0.06 | -0.42 | 0.16 | 0.26 |
| Compound 8 | -0.25 | 0.06 | 0.14 | 0.61^**^ | -0.37 | 0.51^*^ | 0.09 | -0.31 | -0.02 | 0.14 | 0.12 | -0.18 | 0.44 | 0.07 | -0.32 | 0.17 | -0.29 |
| Compound 9 | -0.05 | -0.17 | -0.19 | -0.19 | -0.18 | -0.22 | -0.17 | -0.19 | -0.14 | -0.16 | -0.15 | -0.16 | -0.12 | -0.10 | 0.12 | -0.44^*^ | -0.13 |
| Compound 10 | -0.19 | -0.13 | -0.14 | -0.14 | -0.13 | -0.17 | -0.12 | -0.15 | -0.11 | -0.12 | -0.12 | -0.12 | -0.09 | -0.08 | **0.93^**^** | -0.33 | -0.10 |
| Compound 11 | 0.78^**^ | -0.16 | -0.15 | 0.37 | -0.01 | 0.46^*^ | -0.16 | -0.03 | -0.13 | 0.64^**^ | -0.14 | 0.79^**^ | 0.00 | -0.10 | 0.09 | 0.13 | 0.34 |
| Compound 12 | 0.22 | -0.21 | 0.00 | -0.24 | -0.22 | -0.26 | -0.21 | -0.25 | -0.18 | -0.18 | -0.19 | -0.20 | -0.15 | -0.13 | 0.25 | -0.47^*^ | -0.16 |
| Compound 13 | -0.26 | 0.13 | -0.22 | -0.51^*^ | -0.01 | -0.51^*^ | 0.25 | -0.04 | 0.25 | -0.39 | 0.06 | -0.17 | -0.25 | 0.22 | 0.64^**^ | -0.09 | 0.15 |
| Compound 14 | -0.13 | -0.30 | 0.57^*^ | -0.35 | -0.32 | -0.38 | -0.30 | -0.37 | -0.26 | -0.26 | -0.28 | -0.29 | -0.21 | -0.19 | 0.11 | -0.34 | -0.24 |
| Compound 15 | 0.07 | -0.25 | -0.18 | -0.24 | -0.27 | -0.25 | -0.25 | -0.29 | -0.21 | -0.18 | -0.23 | -0.21 | -0.12 | -0.16 | 0.56^*^ | -0.58^*^ | -0.20 |
| Compound 16 | 0.38 | -0.14 | -0.13 | -0.09 | -0.15 | -0.09 | -0.14 | -0.17 | -0.11 | -0.06 | -0.12 | -0.05 | -0.08 | -0.08 | 0.15 | -0.30 | -0.11 |
| Compound 17 | -0.19 | -0.18 | 0.59^*^ | -0.21 | -0.19 | -0.22 | -0.18 | -0.22 | -0.15 | -0.16 | -0.16 | -0.18 | -0.12 | -0.11 | -0.19 | 0.00 | -0.15 |
| Compound 18 | 0.48^*^ | -0.05 | -0.16 | -0.06 | 0.31 | -0.06 | -0.05 | 0.30 | 0.08 | -0.07 | -0.13 | 0.01 | -0.12 | -0.03 | -0.18 | 0.07 | 0.91^**^ |
| Compound 19 | -0.04 | -0.13 | 0.48^*^ | -0.15 | -0.13 | -0.16 | -0.13 | -0.16 | -0.11 | -0.12 | -0.11 | -0.13 | -0.09 | -0.08 | -0.14 | 0.04 | -0.11 |
| Compound 20 | 0.49^*^ | -0.17 | -0.01 | 0.49^*^ | -0.18 | 0.53^*^ | -0.17 | -0.20 | -0.13 | 0.69^**^ | -0.15 | **0.83^**^** | 0.01 | -0.11 | 0.10 | 0.14 | -0.14 |
| Compound 21 | -0.18 | -0.20 | 0.72^**^ | -0.22 | -0.21 | -0.24 | -0.19 | -0.24 | -0.16 | -0.17 | -0.18 | -0.19 | -0.13 | -0.12 | -0.21 | 0.00 | -0.16 |
| Compound 22 | 0.23 | -0.17 | -0.19 | -0.20 | -0.18 | -0.21 | -0.17 | -0.21 | -0.15 | -0.17 | -0.15 | -0.17 | -0.12 | -0.11 | 0.47^*^ | -0.45^*^ | -0.13 |
| Compound 23 | 0.18 | -0.19 | -0.21 | **0.87^**^** | -0.21 | **0.89^**^** | -0.19 | -0.09 | -0.16 | 0.58^*^ | -0.17 | 0.02 | 0.773^**^ | -0.12 | -0.13 | -0.19 | -0.16 |
| Compound 24 | 0.62^**^ | -0.18 | -0.17 | 0.72^**^ | -0.19 | **0.87^**^** | -0.18 | -0.13 | -0.14 | **0.97^**^** | -0.16 | 0.74^**^ | 0.54^*^ | -0.11 | 0.07 | -0.02 | -0.15 |
| Compound 25 | 0.52^*^ | -0.17 | -0.17 | 0.62^**^ | -0.18 | **0.87^**^** | -0.16 | -0.05 | -0.14 | **0.95^**^** | -0.14 | 0.40 | **0.88^**^** | -0.10 | 0.00 | -0.18 | -0.14 |
| Compound 26 | -0.28 | 0.53^*^ | 0.06 | -0.26 | -0.09 | -0.27 | 0.40 | -0.12 | **0.91^**^** | -0.22 | 0.01 | 0.02 | -0.16 | 0.20 | -0.09 | 0.32 | -0.13 |
| Compound 27 | -0.22 | 0.56^*^ | -0.20 | -0.15 | -0.18 | -0.13 | **0.85^**^** | -0.21 | 0.16 | -0.15 | **0.92^**^** | 0.24 | -0.11 | 0.67^**^ | -0.15 | **0.83^**^** | -0.14 |
| Compound 28 | 0.15 | -0.16 | -0.18 | 0.70^**^ | -0.18 | 0.79^**^ | -0.16 | -0.03 | -0.14 | 0.56^*^ | -0.14 | -0.12 | **0.90^**^** | -0.10 | -0.15 | -0.26 | -0.13 |
| Compound 29 | 0.25 | -0.29 | -0.32 | -0.33 | 0.67^**^ | -0.31 | -0.29 | 0.66^**^ | -0.24 | -0.28 | -0.25 | -0.28 | -0.20 | -0.18 | 0.08 | -0.34 | 0.83^**^ |
| Compound 30 | 0.17 | -0.21 | -0.24 | -0.24 | **0.86^**^** | -0.21 | -0.21 | **0.87^**^** | -0.18 | -0.20 | -0.18 | -0.21 | -0.14 | -0.13 | -0.22 | -0.12 | 0.79^**^ |
| Compound 31 | 0.37 | -0.21 | -0.21 | **0.98^**^** | -0.23 | **0.92^**^** | -0.21 | -0.18 | -0.17 | 0.71^**^ | -0.19 | 0.51^*^ | 0.40 | -0.13 | -0.02 | 0.01 | -0.17 |
| Compound 32 | 0.26 | -0.16 | -0.18 | -0.19 | -0.17 | -0.20 | -0.16 | -0.19 | -0.14 | -0.16 | -0.14 | -0.16 | -0.11 | -0.10 | 0.09 | -0.42 | -0.13 |
| Compound 33 | 0.32 | 0.36 | -0.26 | 0.28 | -0.27 | 0.36 | 0.43 | -0.30 | 0.50^*^ | 0.54^*^ | 0.21 | **0.91^**^** | 0.00 | 0.32 | 0.14 | 0.52^*^ | -0.21 |
| Compound 34 | 0.43 | -0.20 | -0.21 | **0.85^**^** | -0.22 | **0.98^**^** | -0.20 | -0.10 | -0.17 | **0.87^**^** | -0.17 | 0.34 | **0.83^**^** | -0.13 | -0.05 | -0.16 | -0.17 |
| Compound 35 | 0.20 | -0.25 | 0.01 | 0.32 | 0.25 | 0.35 | -0.25 | 0.27 | -0.25 | 0.31 | -0.19 | 0.17 | 0.17 | -0.15 | -0.31 | 0.04 | 0.29 |
| Compound 36 | 0.49^*^ | -0.38 | -0.11 | 0.56^*^ | -0.40 | 0.50^*^ | -0.38 | -0.41 | -0.31 | 0.49^*^ | -0.34 | 0.45^*^ | 0.07 | -0.24 | 0.40 | -0.32 | -0.31 |
| Compound 37 | 0.23 | -0.29 | 0.20 | -0.03 | 0.02 | 0.00 | -0.29 | 0.03 | -0.24 | 0.09 | -0.26 | 0.06 | -0.05 | -0.18 | -0.18 | -0.06 | 0.33 |
| Compound 38 | -0.15 | -0.18 | 0.01 | 0.71^**^ | -0.19 | 0.45^*^ | -0.18 | -0.18 | -0.15 | -0.02 | -0.16 | -0.16 | 0.08 | -0.11 | -0.19 | 0.01 | -0.15 |
| Compound 39 | -0.13 | -0.21 | **0.95^**^** | -0.23 | -0.22 | -0.26 | -0.20 | -0.25 | -0.17 | -0.13 | -0.19 | -0.20 | -0.14 | -0.13 | -0.12 | -0.09 | -0.16 |
| Compound 40 | -0.19 | 0.68^**^ | -0.17 | -0.18 | -0.15 | -0.15 | 0.64^**^ | -0.19 | 0.07 | -0.15 | **1.00^**^** | 0.13 | -0.11 | 0.37 | -0.12 | 0.74^**^ | -0.12 |
| Compound 41 | 0.04 | -0.21 | -0.24 | -0.24 | -0.22 | -0.26 | -0.21 | -0.25 | -0.18 | -0.20 | -0.19 | -0.20 | -0.15 | -0.13 | 0.71^**^ | -0.55^*^ | -0.17 |
| Compound 42 | 0.01 | -0.21 | -0.24 | -0.24 | -0.22 | -0.27 | -0.21 | -0.25 | -0.18 | -0.20 | -0.19 | -0.21 | -0.15 | -0.13 | 0.70^**^ | -0.55^*^ | -0.17 |
| Compound 43 | -0.14 | -0.14 | -0.16 | -0.16 | -0.15 | -0.18 | -0.14 | -0.17 | -0.12 | -0.13 | -0.13 | -0.14 | -0.10 | -0.09 | **0.95^**^** | -0.36 | -0.11 |
| Compound 44 | 0.06 | -0.29 | -0.08 | -0.18 | 0.46^*^ | -0.14 | -0.28 | 0.57^*^ | -0.24 | -0.13 | -0.25 | -0.25 | -0.02 | -0.18 | -0.20 | -0.19 | 0.42 |
| Compound 45 | -0.03 | -0.13 | 0.52^*^ | -0.15 | -0.14 | -0.16 | -0.13 | -0.16 | -0.11 | -0.12 | -0.12 | -0.13 | -0.09 | -0.08 | -0.14 | 0.04 | -0.11 |
| Compound 46 | 0.19 | 0.27 | -0.19 | -0.04 | 0.11 | -0.09 | 0.16 | 0.13 | 0.58^*^ | -0.11 | -0.12 | 0.12 | -0.20 | 0.06 | -0.16 | 0.27 | 0.47^*^ |
| Compound 47 | 0.13 | 0.72^**^ | -0.42 | 0.25 | 0.05 | 0.29 | 0.54^*^ | 0.03 | 0.54^*^ | 0.23 | 0.60^**^ | 0.41 | 0.12 | 0.25 | -0.24 | 0.67^**^ | 0.11 |
| Compound 48 | -0.24 | -0.18 | 0.62^**^ | -0.20 | -0.19 | -0.22 | -0.17 | -0.21 | -0.15 | -0.15 | -0.16 | -0.17 | -0.12 | -0.11 | -0.19 | -0.03 | -0.14 |
| Compound 49 | -0.18 | -0.21 | **0.99^**^** | -0.23 | -0.22 | -0.26 | -0.20 | -0.25 | -0.17 | -0.14 | -0.19 | -0.20 | -0.14 | -0.13 | -0.22 | -0.02 | -0.17 |
| Compound 50 | -0.12 | -0.12 | -0.17 | 0.06 | 0.79^**^ | 0.04 | -0.12 | 0.70^**^ | -0.09 | -0.02 | -0.10 | 0.02 | -0.09 | -0.07 | -0.13 | -0.05 | 0.23 |
| Compound 51 | -0.29 | **0.93^**^** | -0.23 | -0.24 | -0.21 | -0.22 | 0.75^**^ | -0.25 | 0.79^**^ | -0.20 | 0.71^**^ | 0.15 | -0.15 | 0.39 | -0.07 | 0.71^**^ | -0.17 |
| Compound 52 | 0.60^**^ | -0.13 | -0.12 | 0.55^*^ | -0.14 | 0.63^**^ | -0.13 | -0.15 | -0.11 | 0.80^**^ | -0.12 | **0.89^**^** | 0.12 | -0.08 | 0.15 | 0.13 | -0.11 |
| Compound 53 | 0.14 | -0.22 | -0.07 | 0.15 | -0.05 | 0.26 | 0.37 | -0.02 | 0.00 | 0.44 | -0.01 | 0.68^**^ | 0.11 | 0.53^*^ | -0.10 | 0.38 | -0.15 |
| Compound 54 | 0.07 | -0.18 | 0.47^*^ | 0.05 | -0.19 | 0.05 | -0.18 | -0.19 | -0.15 | 0.08 | -0.16 | -0.01 | 0.06 | -0.11 | -0.15 | 0.02 | -0.15 |
| Compound 55 | 0.34 | -0.31 | -0.33 | 0.61^**^ | 0.57^*^ | 0.64^**^ | -0.31 | 0.59^*^ | -0.26 | 0.55^*^ | -0.27 | 0.39 | 0.26 | -0.20 | -0.13 | -0.10 | 0.24 |
| Compound 56 | -0.15 | -0.15 | -0.17 | -0.15 | **0.93^**^** | -0.12 | -0.15 | **0.86^**^** | -0.13 | -0.12 | -0.13 | -0.14 | -0.08 | -0.09 | -0.16 | -0.14 | 0.33 |
| Compound 57 | 0.08 | -0.19 | -0.20 | **0.94^**^** | -0.20 | 0.79^**^ | -0.19 | -0.14 | -0.16 | 0.38 | -0.17 | 0.08 | 0.40 | -0.12 | -0.12 | -0.06 | -0.15 |
| Compound 58 | 0.27 | -0.21 | -0.22 | **0.98^**^** | -0.23 | **0.94^**^** | -0.21 | -0.14 | -0.17 | 0.65^**^ | -0.19 | 0.26 | 0.60^**^ | -0.13 | -0.09 | -0.09 | -0.17 |
| Compound 59 | 0.04 | -0.21 | -0.24 | -0.24 | -0.22 | -0.26 | -0.21 | -0.25 | -0.18 | -0.20 | -0.19 | -0.20 | -0.15 | -0.13 | 0.71^**^ | -0.55^*^ | -0.16 |
| Compound 60 | -0.20 | -0.15 | -0.16 | -0.17 | -0.15 | -0.19 | -0.14 | -0.17 | -0.12 | -0.14 | -0.13 | -0.14 | -0.10 | -0.09 | **0.90^**^** | -0.38 | -0.11 |

**. p<0.01; *. p<0.05; bold labeled number, correlation coefficent r-value > 0.8.
